# Genome-wide association studies of mineral and phytic acid concentrations in pea (*Pisum sativum* L.) to evaluate biofortification potential

**DOI:** 10.1093/g3journal/jkab227

**Published:** 2021-07-08

**Authors:** Sarah Powers, J Lucas Boatwright, Dil Thavarajah

**Affiliations:** Plant and Environmental Sciences, Clemson University, Clemson, SC 29634, USA

**Keywords:** pea, biofortification, genome-wide association study, iron, phosphorus, zinc, phytic acid

## Abstract

Pea (*Pisum sativum* L.) is an important cool season food legume for sustainable food production and human nutrition due to its nitrogen fixation capabilities and nutrient-dense seed. However, minimal breeding research has been conducted to improve the nutritional quality of the seed for biofortification, and most genomic-assisted breeding studies utilize small populations with few single nucleotide polymorphisms (SNPs). Genomic resources for pea have lagged behind those of other grain crops, but the recent release of the Pea Single Plant Plus Collection (PSPPC) and the pea reference genome provide new tools to study nutritional traits for biofortification. Calcium, phosphorus, potassium, iron, zinc, and phytic acid concentrations were measured in a study population of 299 different accessions grown under greenhouse conditions. Broad phenotypic variation was detected for all parameters except phytic acid. Calcium exhibited moderate broad-sense heritability (H^2^) estimates, at 50%, while all other minerals exhibited low heritability. Of the accessions used, 267 were previously genotyped in the PSPPC release by the USDA, and we mapped the genotyping data to the pea reference genome for the first time. This study generated 54,344 high-quality SNPs used to investigate the population structure of the PSPPC and perform a genome-wide association study to identify genomic loci associated with mineral concentrations in mature pea seed. Overall, we were able to identify multiple significant SNPs and candidate genes for iron, phosphorus, and zinc. These results can be used for genetic improvement in pea for nutritional traits and biofortification, and the candidate genes provide insight into mineral metabolism.

## Introduction

In the past few decades, the plant breeding field has primarily focused on increasing yield in the staple cereal crops rice (*Oryza sativa* L.), wheat (*Triticum aestivum* L.), and maize (*Zea mays* L.), which now account for most calories consumed worldwide (FAO 2004). While cereals are good sources of carbohydrates, they have inadequate protein and micronutrient levels to sustain a healthy diet, which may contribute to micronutrient deficiencies termed “hidden hunger” (Pingali 2012). Biofortification is a strategy to increase the nutritional quality of food crops through agronomic practices, conventional plant breeding, and biotechnological approaches (Welch and Graham 2004; Foyer *et al.* 2016). Several successful vitamin and micronutrient biofortified varieties have been developed and released for common bean (*Phaseolus vulgaris* L.), cassava (*Manihot esculenta* L.), cowpea (*Vigna unguiculata* L.), lentil (*Lens culinaris* L.), maize, pearl millet (*Pennisetum glaucum* L.*)*, sorghum (*Sorghum bicolor* L.), sweet potato (*Ipomoea batatas* L.), and wheat, with the most notable release being pro-vitamin A Golden rice (Ye *et al.* 2000; Saltzman *et al.* 2013). However, minimal plant breeding or biofortification research has been dedicated to pea (*Pisum sativum* L.) despite its potential to alleviate hidden hunger.

Pea is a cool-season crop grown primarily to benefit soil health, nitrogen-fixing capabilities, nutrient-dense seed, and affordability for consumers (Amarakoon *et al.* 2012; Foyer *et al.* 2016; Stagnari *et al.* 2017). Pea provides superior amounts of protein than cereals and has a greater protein concentration than chickpea (*Cicer arietinum* L.) and cowpea (Iqbal *et al.* 2006). In addition, pea is rich in prebiotic carbohydrates, fiber, and micronutrients, especially iron (Fe) and zinc (Zn), making it an ideal candidate for biofortification. Pea breeding efforts have lagged behind those for cereals, so the potential for increasing nutritional concentrations in pea seed is largely unexplored (Amarakoon *et al.* 2012; Foyer *et al.* 2016; Rehman *et al.* 2019) . A few studies investigating nutritional variation and the underlying genetic mechanisms have been reported in pea. Still, these studies have utilized small populations, were conducted before the release of the pea reference genome, and offer little insight into candidate genes involved in mineral concentration in the seed (Diapari *et al.* 2015; Ma *et al.* 2017; Gali *et al.* 2018, 2019; Dissanayaka *et al.* 2020; Jha *et al.* 2020). Pea is also low in phytic acid (IP6), an antinutrient that decreases micronutrient bioavailability to humans (Raboy 2003). Low phytic acid (*lpa*) mutants have been created in pea (Raboy *et al.* 2000; Warkentin *et al.* 2012), but whether traditional breeding efforts can affect IP6 concentrations remains to be investigated. Thus, genomic studies in larger, diverse populations could evaluate the feasibility of biofortification in pea.

In 2019, Kreplak *et al.* published the first sequence of the pea genome. The genome (2*n* = 14) is 4.45 Gb in size and largely repetitive, both of which contribute to the lack of genomic resources for pea (Kreplak *et al.* 2019). Pea is a self-pollinating species, resulting in high degrees of inbreeding and a lack of genetic diversity compared to other crops, such as maize or wheat. The USDA Pea Single Plant Plus Collection (PSPPC) was assembled by Holdsworth *et al.* in 2017 and specifically designed to capture genetic diversity in pea for genomic-assisted breeding. The PSPPC comprises 431 accessions of pea that have been genotyped through genotype by sequencing (GBS), and Holdsworth *et al.* (2017) also genotyped 25 accessions of *Pisum fulvum*, a relative of *P. sativum*, that can be used in diversity analysis (*n* = 456). The collection constitutes multiple subpopulations (*P. sativum* subsp. *elatius*, *P. sativum* subsp. *abyssinicum*, and *P. sativum* subsp. *sativum*), as well as accessions from Central Asia, representing a novel source of alleles (Holdsworth *et al.* 2017). To our knowledge, no genome-wide association studies (GWAS) for nutritional traits have explicitly used the PSPPC at the time of this publication; thus, the potential for the PSPPC in biofortification research has not been explored. A GWAS makes statistical associations between a phenotype and single nucleotide polymorphisms (SNPs) present in the study population to identify genomic regions and genes associated with the trait of interest (Huang and Han 2014). As the PSPPC was designed to encompass the genetic diversity of pea and is a large collection of pea germplasm available for public use, the PSPPC is ideal for GWAS to investigate the genetic basis of seed nutrients concentrations in pea.

By phenotyping seed mineral concentrations in the PSPPC and combining the results with the GBS data, genetic variation can be analyzed to aid in biofortification research for pea. In addition, alignment of the PSPPC GBS data to the reference genome and provision of the assembled variant call format (VCF) and HapMap files allows researchers to perform GWAS and identify associated genomic loci using the publicly available annotation. Here, we identified significant SNPs and candidate genes associated with mineral concentration. These results can be used to understand mineral metabolism and accumulation in the seed. Overall, this study aims to evaluate the genetic diversity and biofortification potential of pea and identify genomic loci related to nutrient concentrations in mature pea seeds that could be used to breed biofortified pea varieties.

## Materials and methods

### Plant material and growth conditions

A total of 299 *P. sativum* accessions comprised 267 genotyped PSPPC accessions, 29 nongenotyped accessions, commercial cultivars Cameor, Hampton, and CDC Bronco were planted in a complete randomized design with three replicates at the Clemson University Greenhouse Complex, Clemson, SC, USA. Two plants per accession were planted in 6″ pots and grown in potting soil (SunGro Professional Growing Mix SKU: SUGR2375003; pH 6.4) under conditions of 16 hours day and temperatures of 20–22/18°C day/night. All pots were watered to 70–80% pot capacity. Plants were not inoculated with rhizobia, as they were supplied with adequate nitrogen, and nitrogen fixation was not necessary. A week after planting, all plants were given 2.84 g of osmocote (14-14-14); an additional starter of Peter’s Professional 20-20-20 fertilizer was given 5 days later. Plants were continuously fertilized with Peter’s Professional fertilizer every 2 weeks until they were physiologically mature, approximately 90 days after planting, with the mature seed then harvested.

### Mineral analysis

The harvested mature seeds were ground into a fine powder using a KitchenAid coffee bean grinder and stored at 4°C until analyzed. To prepare samples for analysis, 200 mg of seed were digested overnight in 4 mL of concentrated nitric acid (70% HNO_3_). The seed samples were then heated to 150°C for 2 hours, with 4 mL of hydrochloric acid (70% HCl), then added to the solution and heated for an additional 1 hour. The digested solution was then filtered through Whatman paper (20–25 µm) and diluted to 10 mL with deionized H_2_O. Mineral concentrations of Ca, P, K, Fe, and Zn were determined by inductively coupled plasma emission spectrometry (ICP-OES; ICP-6500 Duo, Thermo Fisher Scientific, Pittsburg, PA, USA). Standards made from a 1000 mg L^−1^ stock solution were serially diluted to produce calibration curves from 0.5 to 5.0 mg L^−1^. Measurements using this method were validated using lentil and peach as standard references. Moisture content was analyzed from a random subsample of 28 samples for each replicate, with data averaged to obtain the moisture content for the specific replicate.

### Phytic acid (IP6) analysis

Seed samples were prepared using the modified IP6 extraction from Talamond *et al.* (2000) and Thavarajah *et al.* (2009). A 100-mg sample of finely ground seed was weighed into a 15-mL conical tube (17 ± 120 mm) with a fitted cap. Then 10 mL of 0.5 M HCl were added to the tube, which was submerged into boiling water (∼100°C) for 5 minutes. The solution was centrifuged for 3 minutes, and the supernatant was transferred into a separate tube. The IP6 was demultiplexed with the addition of 1.5 mL of 12 M HCl. High-performance liquid chromatography with a conductivity detector was used for IP6 analysis (ICS-5000 Dionex, Sunnyvale, CA, USA). The IP6 was separated with an Omnipac Pax-100 guard column (8 µm) and quantified by conductivity detection. The solvents used for gradient elution were 130 mM sodium hydroxide (A), deionized water-isopropanol (50:50, v/v) (B), and water (C). The flow rate of the gradient elution was 1.0 mL min^−1^ with a total run time of 10 minutes. Retention time and peak area were used to quantify the IP6 in the seed samples. IP6 standards from 10 to 500 mg L^−1^ were used for calibration curves, with the detection limit set at 5 mg L^−1^. The error tolerance was <0.1% for all laboratory samples. The IP6 phosphorus concentration was calculated using the weight ratio of P atoms per molecule of IP6 (1:3.56).

### Phenotypic and statistical analysis

The distributions of mineral concentration for each accession were visualized using JMP Pro 14 software (SAS Institute Inc., Cary, NC, USA), and accessions containing outliers in one or more replicates were identified as 1.5 times the interquartile range above the upper quartile and below the lower quartile and excluded from further analysis. In addition, outliers were defined as accessions with mean values greater than three standard deviations away from the population mean and excluded from the final dataset (Supplementary File S1). After outliers were removed, broad-sense (H^2^) heritability estimates for each mineral were obtained in JMP by determining the ratio of variance due to accessions divided by the total variance for the phenotype (V_G_/V_P_). For correlation analysis, accessions with missing data in one or more minerals were excluded, and Pearson’s r estimates were calculated in Python and assigned to correlation coefficients (ρ). IP6 was excluded from correlation analysis as it forced the exclusion of numerous accessions that negatively affected the analysis.

### Genotyping and data processing

The PSPPC and *P. fulvum* (*n* = 456) accessions were previously genotyped by Holdsworth *et al.* (2017), using the GBS method described in Elshire *et al.* (2011). All raw sequencing reads were retrieved from the National Center for Biotechnology Information (NCBI) (http://www.ncbi.nlm.nih.gov/bioproject/379298). The raw reads were aligned to the current pea reference genome (https://urgi.versailles.inra.fr/Species/Pisum) using the Burrow-Wheeler aligner (Li and Durbin 2010), with a mean accuracy of 99% of each read aligned. SNPs were called using the current version of the Genome Analysis Toolkit (GATK) (https://gatk.broadinstitute.org/) and hard filtered for quality, missing data, and minor allele frequency (>0.05) using GATK and BCFtools, prior to genomic analysis. Beagle version 5.1 was used to impute missing genotype data in the VCF file assembled from GATK. The final VCF file was converted into the HapMap format using Tassel version 5.2.52.

### Population structure and GWAS

Using the final imputed and filtered VCF file, admixture of the PSPPC+*P. fulvum* population was estimated using ADMIXTURE (Alexander and Lange 2011) and graphed using R. ADMIXTURE produces a Q matrix containing estimates of ancestry for each individual tested. The corresponding Q matrix with the lowest cross-validation error was chosen as the most representative of the study population, which was at *K* = 11, corresponding to 11 distinct subpopulations. The species information was obtained from supplementary information from Holdsworth *et al.* (2017) and assigned to the corresponding accession in the Q matrix. Accessions with unavailable species information were annotated as *P. sativum* in this study for both admixture analysis and principal components analysis (PCA). The Q matrix was then sorted by the ancestry coefficients for each subpopulation, assigning individuals with coefficients >50% to the corresponding subpopulation (Boatwright *et al.* 2021). Principal components (PCs) were calculated during analysis with GAPIT (Wang and Zhang 2020); the first two PCs were graphed using R and assigned a color based on available species information.

In addition, analysis of variance (ANOVA) testing was conducted to determine if there were any batch effects associated with the date of sample digestion or the date of analysis (Supplementary File S2). If significant effects were observed, best linear unbiased predictors (BLUPs) were calculated to incorporate each source of variation for each nutritional trait to be used as the phenotype instead of the mean. The BLUP models were calculated using the lme4 package in R (Bates *et al.* 2015) and were fit for each accession using the following form:

y=(1|Taxa)+(1|Replicate)+(1|Dateofdigestion)+(1|Dateofanalysis)

where *y* is the observed mean and **Taxa, Replicate, Date of digestion, Date of analysis** were included as random effects.

GAPIT was used to perform a GWAS on mineral and IP6 concentrations of mature pea seed using the HapMap file. The default GAPIT parameters were used, as well as a model selection with Bayesian Information Criterion (BIC), which determines the degree of population structure that should be accounted for in a model to avoid overfitting. The BIC analysis determined that the inclusion of PCs was not necessary for any of the models. A mixed linear model with a kinship matrix was selected for analysis to account for population stratification. The mixed linear models were fit using the following form:

y=Xβ+Zu+e

where *y* is a vector of observed phenotypes; β is an unknown vector containing fixed effects that account for the genetic marker, population structure (Q), and intercept; u is an unknown vector of random additive effects from background QTLs and individuals; X and Z are the known design matrices, and **e** is the unobserved vector of residuals according to the GAPIT user manual.

The Bayesian-information and Linkage-disequilibrium Iteratively Nested Keyway (BLINK) method was also utilized for GWAS of the nutritional traits, as it has high statistical power and does not assume that causal genes are distributed normally across the genome, which can lead to false positives and exclusion of causal genes (Huang *et al.* 2019). BLINK uses BIC to exclude markers based on linkage disequilibrium (LD) so that only the most significant markers are reported (Huang *et al.* 2019). The BLINK models were fit using the following form:

$$y=s_{i}+S+e$$

where *y* is a vector of observed phenotypes; *s_i_* is a testing marker; *S* is a pseudo quantitative trait nucleotide (QTN), and *e* is the unobserved vector of residuals according to the GAPIT user manual. Analysis was also conducted using multiple loci mixed models (MLMMs) and compressed mixed linear models (CMLMs) (Supplementary File S2). A Bonferroni correction was used to avoid false positives and identify significant SNPs (α = 0.05) for each trait. The Bonferroni correction was calculated as −log_10_(0.05/*n*), where *n* equals the number of SNPs used in the GWAS for each mineral.

### Linkage disequilibrium and identification of candidate genes

LD decay was estimated for each chromosome using PopLDdecay (Zhang *et al.* 2019) using MAF = 0.05 (Supplementary File S2), and the LD of significant SNPs was estimated using Plink 1.90 b with an LD window of 1000 kb. Genes in local LD with each significant SNP were identified using custom Python scripts and considered to be candidate genes for mineral concentration in mature pea seed. Identification of gene function was obtained using PulseDB of the *P. sativum* v1a genome (https://www.pulsedb.org/jbrowses) or through the National Center of Biotechnology Information (NCBI) BLAST tool with *Medicago truncatula* and *Glycine max* as the reference organisms.

### Data availability

All scripts, data, and the VCF and HapMap used for this project are available at https://github.com/selizpowers/GWAS. All raw GBS data for the PSPPC are accessible at https://www.ncbi.nlm.nih.gov/bioproject/PRJNA379298.

Supplementary material is available at *G3* online.

## Results

### Mineral analysis of *P. sativum* accessions

**Figure 1:**
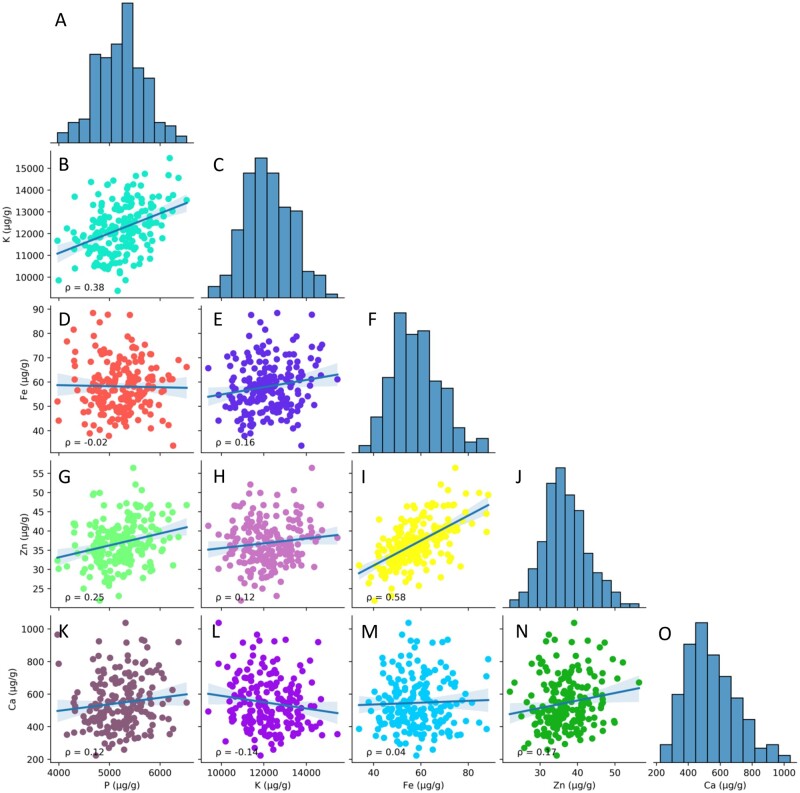
Distribution and correlations of minerals in mature pea seed (*n* = 222). The distributions of mean concentrations for P (A), K (C), Fe (F), Zn (J), and Ca (O) are depicted in each histogram. The scatter plots show Pearson coefficients (ρ) between minerals: P *vs* K (B), P *vs* Fe (D), P *vs* Zn (G), P *vs* Ca (K), K *vs* Fe (E), K *vs* Zn (H), K *vs* Ca (L), Fe *vs* Zn (I), Fe *vs* Ca (M), and Zn *vs* Ca (N). The blue shaded regions on the scatterplots represent the 95% confidence intervals for each correlation. Accessions missing data for one or more minerals were excluded.

The phenotypic variation of mineral and IP6 concentrations is broad across all accessions (Table 1) (Figure 1, A, C, F, J, and O). Pea also provides a good amount of the recommended daily allowance (RDA) for all minerals (32–76%) except Ca (5%). Broad-sense (H^2^) heritability estimates are low for most minerals, except for Ca (50%) (Table 1). In addition, analysis using Pearson’s correlation coefficient (ρ) reveals low to moderate correlations among various nutrients, with the strongest (ρ > 0.5) correlation noted between Fe and Zn concentrations (ρ = 0.58). A moderate correlation (0.3 < ρ < 0.5) was observed between P and K (ρ = 0.38).

**Table 1:** Ranges, means, recommended daily allowance percentage (%RDA), and broad-sense heritability (H^2^) estimates of mature pea seed concentrations of each nutrient for all accessions

Mineral	Range (µg/g)	Mean (µg/g)*a*	RDA*b* (%)	H^2^ (%)
P (*n* = 268)	3,973.8–6,623.8	5,269.4 ± 29.67	75	23
K (*n* = 279)	9,375.1–15,809.2	12,286.6 ± 71.44	36–47	15
Fe (*n* = 247)	30.7–88.4	58 ± 0.69	32–73	30
Zn (*n* = 269)	22–52.4	37.6 ± 0.34	34–48	28
Ca (*n* = 245)	223.5–1,038.4	558.46 ± 10.4	5	50
IP6 (*n* = 184)	9.7–13.8	11.71 ± 0.05	—	—

a Mean concentration presented for three replicates ± standard error.

b %RDA based on mean mineral concentration for males and females ages 31–50 according to NIH guidelines (https://ods.od.nih.gov/HealthInformation/Dietary_Reference_Intakes.aspx).

### Population structure analysis of PSPPC+*P. fulvum* collection and study population

**Figure 2:**
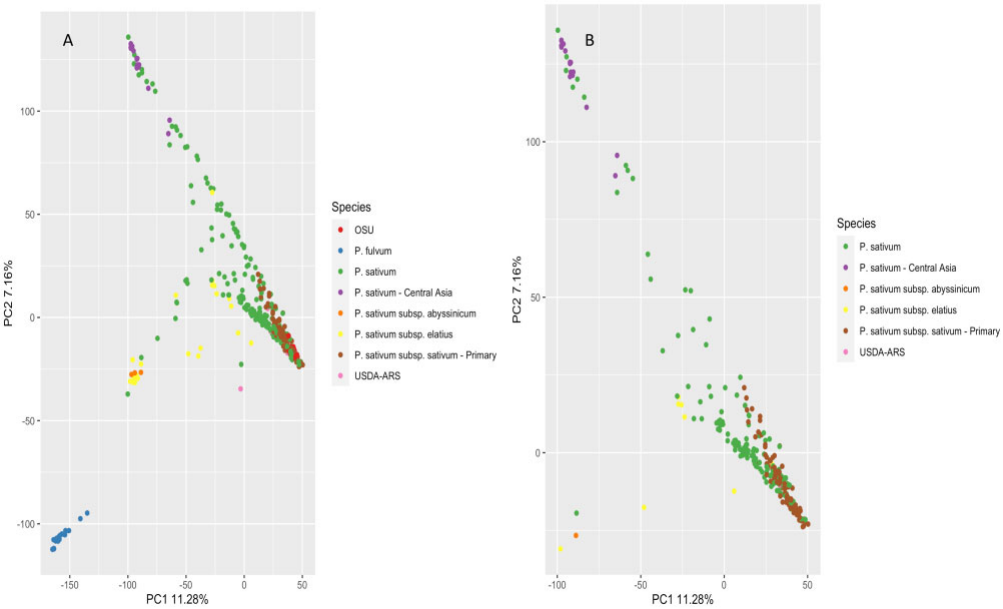
Principal component analysis (PCA) of the PSPPC and the accessions used for GWAS. (A) PCA plot for PSPPC*+P. fulvum* population (*n* = 456), with each colored dot corresponding to a different subpopulation of PSPPC*+P. fulvum*. (B) PCA plot showing the subpopulations of the accessions used in the GWAS for nutrient concentration (*n* = 267). Accessions from the OSU and *P. fulvum* subpopulations were not included in the study population. Accessions with unavailable population information were labeled as *P. sativum.*

**Figure 3:**
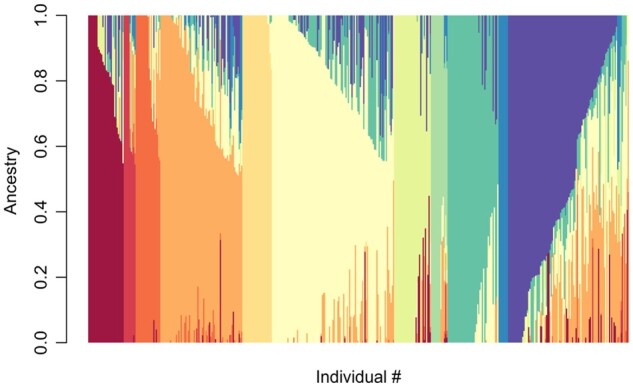
Genome-wide, population admixture analysis of the PSPPC+*P. fulvum* population. The individuals are shown as vertical bars along the *x*-axis and have been given a unique color(s) based on the proportion of estimated ancestry (*y*-axis) for each distinct ancestral population (*K* = 11). Accessions with unavailable population information were labeled as *P. sativum*.

To determine population structure in the GWAS, as well as compare results to the previous *de novo* assembly of the PSPPC, admixture analysis, and PCA were performed on the reference-based alignment of the PSPPC population as well as the study population (Figures 2 and 3). The first two PCs measured in GAPIT each account for 11.28 and 1.89% of the variation, respectively (Figure 2A) with the first PC accounting for less variation than the estimate reported in Holdsworth *et al.* (2017). In addition, most of the accessions appearing in the PSPPC and the study population appear to cluster in the same area across PC1 and PC2 (OSU, *P. sativum*, *P. sativum abyssinicum*, USDA-ARS, and *P. sativum* subsp. *sativum–*Primary), while the accessions assigned to *P. sativum*–Central Asia and *P. sativum* subsp. *elatius* appear to exhibit some population differentiation based on their separation from the main breeding germplasm (Figure 2, A and B). The wild species *P. fulvum* is the most different from the other species, forming a single separate cluster (Figure 2A). These results are supported by the previous report from Holdsworth *et al.* (2017). Analysis of population admixture, however, identified 11 ancestral subpopulations within the PSPPC (Figure 3). Finally, despite *P. sativum* being a highly inbred species, certain accessions within the PSPPC have diverse ancestral backgrounds based upon subpopulation admixture (Figure 3).

### GWAS of mineral and IP6 concentrations in mature *P. sativum* seed

**Figure 4:**
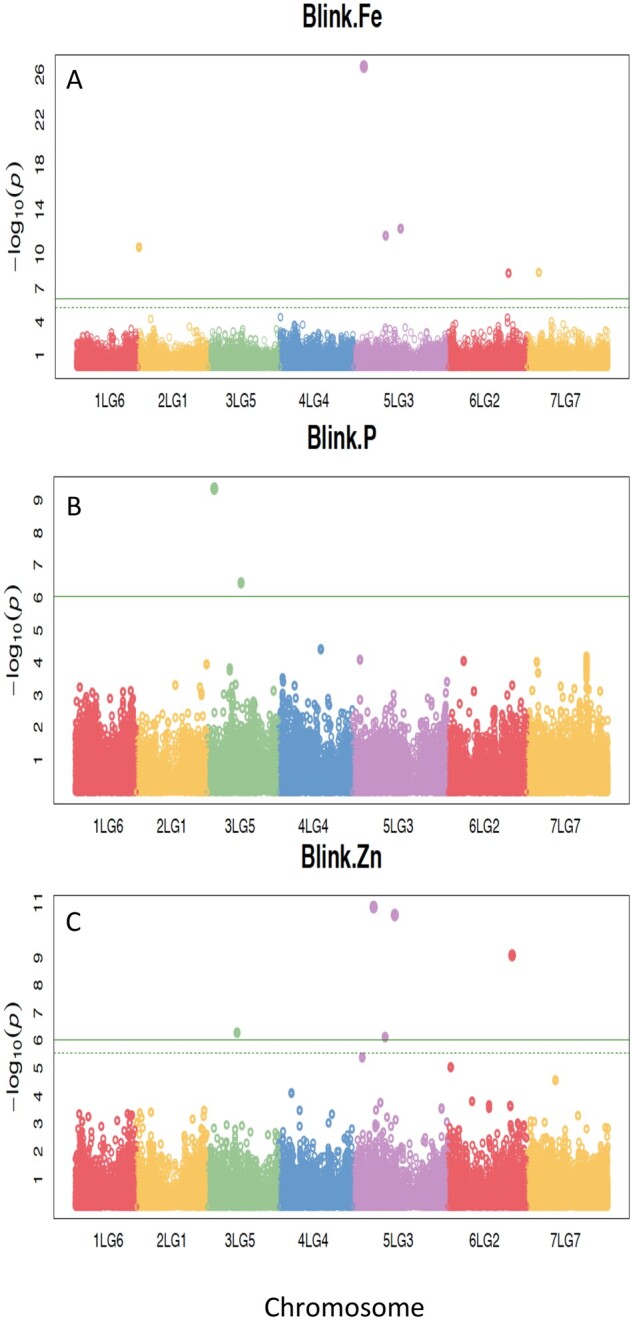
Manhattan plots for Fe, P, and Zn from the BLINK model. Significant SNPs for Fe (A), P (B), and Zn (C) were identified across the pea genome. The −log_10_*P*-values (y-axis) are plotted against the position of each chromosome (*x*-axis), where each circle represents an SNP.

To identify SNPs associated with nutritional traits in mature pea seed, GWAS was performed for several minerals and IP6. Only 267 accessions from the study population (*n* = 299) were genotyped, so the GWAS population for each mineral was as follows: P (*n* = 247), Ca (*n* = 229), K (*n* = 257), Zn (*n* = 248), Fe (*n* = 225), and IP6 (*n* = 175). Phenotypes considered outliers were removed from the GWAS, as were any accessions that did not have available GBS data, resulting in the different population sizes between minerals. All GBS data for the PSPPC+*P.fulvum* (*n* = 456) were used to make both the final VCF and HapMap files to call the maximum number of SNPs. In total, 319,141 SNPs were generated, of which 54,344 high-quality biallelic SNPs were used for the GWAS. A mixed linear model, as well as a BLINK model, was used to map BLUPs of each trait to the *P. sativum* v1a genome. The LD of each chromosome decayed rapidly, which is supported by previous studies in pea (Gali *et al.* 2019; Beji *et al.* 2020). For SNPs, LD was considered to decay at *r*^2^<0.1, and local LD for the significant SNPs of each trait was estimated to be 220, 304, and 211 kb for Fe, P, and Zn, respectively. Significant SNPs were identified from the BLINK model for Fe, P, and Zn across all chromosomes (Figure 4), with Fe and Zn significant SNPs present on chromosome 5 and P exhibiting significant SNPs on chromosome 3. These SNPs were in local LD with multiple candidate genes (Table 2).

**Table 2:** Candidate genes in local LD with the most significant SNPs for mineral seed concentration identified through GWAS

SNP_GROUP	*P*-value	LD block	Mineral	CHR	SNP_POS	GENE_ID	INFO
S5LG3_65230721	1.9e-27	65010721–65450721	Fe	5	65230721	Psat5g034720	ABC1 family mRNA
—	—	—	—	—	—	Psat5g034760	GAGA binding protein-like famly mRNA
—	—	—	—	—	—	Psat5g034800	Inorganic pyrophosphatase 2*a*
—	—	—	—	—	—	Psat5g034840	Protein phosphatase 2 C
—	—	—	—	—	—	Psat5g034880	eIF2A
—	—	—	—	—	—	Psat5g034920	Twin BRCT domain
—	—	—	—	—	—	Psat5g034960	Dicer dimerisation domain
—	—	—	—	—	—	Psat5g035000	Pentotricopeptide repeat- containing protein
—	—	—	—	—	—	Psat5g035040	Unknown
—	—	—	—	—	—	Psat5g035080	Carbon-nitrogen hydrolase
S3LG5_44073760	4.3e-10	43769760–44377760	P	3	44073760	Psat3g019520	WRKY DNA-binding domain
—	—	—	—	—	—	Psat3g019560	Unknown
—	—	—	—	—	—	Psat3g019600	PAPA-1 like conserved region
—	—	—	—	—	—	Psat3g019640	CP12 domain
—	—	—	—	—	—	Psat3g019680	PPR repeat family
—	—	—	—	—	—	Psat3g019720	Serine carboxypeptidase
—	—	—	—	—	—	Psat3g019760	Fact complex subunit (SPT16/CDC68)
—	—	—	—	—	—	Psat3g019800	Prolyl oligopeptidase family
—	—	—	—	—	—	Psat3g019840	GDSL/SGNH-like Acyl-Esterase family found in Pmr5 and Cas1p
—	—	—	—	—	—	Psat3g019880	SET domain
S5LG3_128102170	1.5e-11	127891170–128313170	Zn	5	128102170	Psat5g070360	COP1 interacting protein 7*a*
—	—	—	—	—	—	Psat5g070400	SET domain
—	—	—	—	—	—	Psat5g070440	Peptidase family C78
—	—	—	—	—	—	Psat5g070480	ABC transporter transmembrane region
—	—	—	—	—	—	Psat5g070520	Unknown

a Gene INFO was obtained through BLAST with *Medicago truncatulata* to infer gene function

## Discussion

Pea is an important crop for both sustainable agriculture and human nutrition, as it improves soil health, fixes nitrogen from the atmosphere, and produces a nutrient-dense staple that is affordable for consumers of all socioeconomic status (Amarakoon *et al.* 2012; Foyer *et al.* 2016; Stagnari *et al.* 2017). Pea is high in Fe and Zn, representing two of the most common micronutrient deficiencies worldwide (Bailey *et al.* 2015). Biofortification is a plant breeding strategy employed in pea to select and breed varieties with increased nutritional value to alleviate hidden hunger; however, genomic resources required for nutritional breeding have lagged in pea compared to other crops. The recent release of the *P. sativum* reference genome (Kreplak *et al.* 2019) and the development of the PSPPC (Holdsworth *et al.* 2017) present exciting new opportunities to address nutritional breeding objectives in pea breeding programs around the world.

The pea accessions used in the study population have broad ranges of concentrations for all minerals, with little variability in IP6 (Table 1). All ranges and means are like the findings in previous pea research; however, accessions in the PSPPC have some of the highest Fe concentrations reported (Amarakoon *et al.* 2012; Ray *et al.* 2014; Diapari *et al.* 2015; Bangar *et al.* 2017). Accessions PI_272171, PI_505144, and PI_274307 have the highest mean Fe concentrations at 88.4, 87.7, and 87.6 µg/g, respectively (Supplementary File S1). Accession PI_272171 also has one of the highest Zn concentrations (49.3 µg/g), demonstrating the strong correlation between Fe and Zn (Figure 1I). Positive correlations have been observed for seed Fe, and Zn concentrations in rice and wheat (Sperotto *et al.* 2010; Morgounov *et al.* 2007), and positive correlations between bioaccessible Fe and Zn in cowpea have also been observed (Coelho *et al.* 2021). As the pea accessions in the study are low in IP6 (Table 1) (Amarakoon *et al.* 2012), these results suggest that the Fe and Zn in pea are bioavailable but will need to be confirmed in separate experiments. In addition, Fe (H^2^ = 30%) has the second-highest heritability estimate among minerals after Ca (H^2^ = 50%) (Table 1), so selecting for increased Fe concentration in the seed may positively improve Zn concentration. The moderate heritability of Ca means that trait improvement could be possible using the high Ca accessions, as the study population can only provide an estimated 5% of the RDA of 1000 mg d^−1^. Moderate correlations are noted between P and K (Figure 1B), and these interactions have been reviewed in previous research (Xia and Xiong 1991; Blair *et al.* 2013). All other minerals and IP6 have low heritability, suggesting these traits are predominately influenced by the growing environment rather than genetic factors.

In terms of pea biofortification, these results suggest that breeding for increased mineral concentration cannot be accomplished by phenotyping the seed concentration alone, especially as narrow-sense heritability has been reported to be lower than broad-sense heritability estimates (Banerjee *et al.* 2012; Messiaen *et al.* 2012). Furthermore, this study was conducted under greenhouse conditions, which is a controlled environment, which also indicates heritability estimates may be even lower for these minerals under field conditions, where the environment is variable. Another limitation of this experiment is that it utilized three replicates with two plants for each accession; typically, a GWAS would be conducted on phenotypic data gathered from 4 to 6 field trials, with multiple replicates and plants. Thus, further analysis across different environments and field seasons is necessary to evaluate this study population's biofortification potential accurately. Quantifying alternative phenotypes, such as nutrient uptake from the soil and subsequent remobilization to the mature seed, may improve nutritional quality and provide additional biofortification targets that may be more successful in breeding programs (White and Broadley 2005; Ariza-Nieto *et al.* 2007; Rehman *et al.* 2019).

The PSPPC is a collection of pea germplasm specifically assembled and genotyped to represent the genetic diversity within *P. sativum* as a source of novel alleles for breeding programs (Holdsworth *et al.* 2017). The PCA of the PSPPC (Figure 2A) is consistent with the PCA reported in Holdsworth *et al.* (2017), with all germplasm apart from *P. fulvum*, *P. sativum*–Central Asia, and *P. sativum* subsp. *elatius* clustering together across the first two PCs. Further analysis also showed that *P. sativum*—Central Asia separates further from the breeding germplasm when plotting PC1 against PC3 (Supplementary File S2). Overall, the PCA plots indicate that *P. sativum*–Central Asia and *P. sativum* subsp. *elatius* accessions are the most divergent from the other accessions in the diversity panel; *P. sativum*–Central Asia appears to have little within group diversity while *P. sativum* subsp. *elatius* is a diverse subpopulation (Figure 2, A and B, Supplementary File S2). In addition, the admixture analysis (Figure 3) revealed that the PSPPC has 11 distinct subpopulations with diverse ancestral backgrounds and that there is substantial within-group diversity among the subpopulations. While ancestral and genetic variation exist in the PSPPC, the addition of more diverse accessions could improve the breeding and association mapping potential of the PSPPC.

The PSPPC is also intended to be used as germplasm to improve trait mapping and genomic-assisted breeding in pea (Holdsworth *et al.* 2017). At the time of the PSPPC publication, the *P. sativum* reference genome was unavailable, thus all GBS data were utilized in a *de novo* assembly. As GBS data produce short reads, *de novo* assembly cannot scaffold across the large, highly repetitive sequences of pea, resulting in gaps, missing data, and an incomplete assembly that is not truly representative of the genome (Liao *et al.* 2019). This study is the first to align the PSPPC GBS data to the reference genome to produce a robust GWAS in pea. This study is also significant in pea nutritional research, as it has the largest study population used in any GWAS related to nutritional traits with the largest number of high-quality SNPs (Gali *et al.* 2019; Dissanayaka *et al.* 2020; Jha *et al.* 2020). Both the VCF and HapMap files can be used by other pea researchers, and the addition of SNPs from other genotyped lines will greatly improve GWAS resolution and accuracy in pea research.

Using GWAS with a BLINK model, we were able to identify six significant SNPs for Fe on chromosomes 2, 5, 6, and 7; two significant SNPs for P located on chromosome 3; and five significant SNPs for Zn on chromosomes 3, 5, and 6 (Figure 4). In addition, a peak on chromosomes 1 and 3 was also identified for Ca, and K concentration, respectively, and significant SNPs would likely be identified in that chromosomal region if more samples were included to increase statistical power (Supplementary File S2). Multiple candidate genes were identified in local LD with the most significant SNP (*P* < 9.2 × 10^−7^) for Fe, P, and Zn (Table 2). Of the 10 candidate genes associated with SNP S5LG3_65230721 for Fe, *Psat5g034720* was identified as an ATP-binding cassette 1 (ABC1) family mRNA, which is a gene family that’s involvement has been observed in the assembly of Fe-S clusters and iron homeostasis in Arabidopsis (*Arabidopsis thaliana*) (Moller *et al.* 2001; Xu and Moller 2004; Xu *et al.* 2005; Li and Lan 2017). Another study in Arabidopsis found that mutants that lacked two ABC1 protein kinases accumulated increased amounts of ferritin and superoxides across tissues and demonstrated reduced tolerance for oxidative stress, providing further evidence for the roles of the ABC1 gene family in relation to iron metabolism and stress response (Manara *et al.* 2014). Of the five candidate genes identified for Zn, *Psat5g070480* was also identified as a transmembrane ABC transporter in LD with SNP S5LG3_128102170. ABC transporters have been implicated in Zn homeostasis based on gene ontology analysis in Arabidopsis (Hall and Williams 2003; Broadley *et al.* 2007). An additional ten candidate genes were identified in local LD with SNP S3LG5_44073760 related to P seed concentration. The gene *Psat3g019520* corresponds to a WRKY DNA-binding domain, and WRKY transcription factors are well characterized in their involvement in plant stress response (Bakshi and Oelmüller 2014; Jiang *et al.* 2017). A study in rice found that overexpression of OsWRKY74 increased root and shoot biomass, as well as P concentration in rice grown under P deficient conditions, implicating roles of WRKY transcription factors in modulating P uptake and translocation from the soil (Dai *et al.* 2016). Another study in Arabidopsis found that AtWRKY42 is involved in P homeostasis by regulating the expression of *PHO1* and *PHT1* in response *P* availability in the soil (Su *et al.* 2015). More studies are necessary to elucidate the role of these candidate genes on mineral seed concentration, such as those generating knock-out mutants for these candidate genes to determine their effects on nutrient metabolism and to observe how mineral concentration changes in their absence.

## Conclusions

In summary, this study shows significant variation in pea in mineral concentrations but minimal variation for IP6. Mineral concentrations appear to be predominately influenced by environmental factors, except Ca, which shows moderate H^2^ estimates. These findings suggest alternative phenotypes besides seed concentration should be considered for biofortification research in pea, such as those that consider mineral uptake, mobilization, and accumulation of final mineral concentration. In addition, the PSPPC is genetically diverse and a valuable resource for genomic research in pea, as it has been previously genotyped, is publicly available, and incorporates novel alleles, which is useful to investigate the genetic basis of many agronomic and nutritional traits. Finally, candidate genes for Fe, P, and Zn concentration have been identified through GWAS, and their roles could be further characterized through additional molecular studies via knockouts or gene overexpression experiments. Overall, this research contributes to our understanding of nutritional traits and the future of biofortification and genomic research in pea.

S.P. is a doctoral student with D.T. who developed the experiment design, conducted the research, data analysis, and manuscript preparation. J.L.B. advises S.P. on genomic analysis and manuscript preparation. D.T. is the project P.I., mentoring S.P. on the study and manuscript preparation.

## Funding

Funding support for this project was provided by the Organic Agriculture Research and Extension Initiative (OREI) (award no. 2018-51300-28431/proposal no. 2018-02799) of the United States Department of Agriculture, National Institute of Food and Agriculture, and the USDA-ARS Pulse Health Initiative.

## Conflicts of interest

None declared

## Supplementary Material

jkab227_Supplementary_Data
