# Supplementary material for: Genome-wide association studies of mineral and phytic acid concentrations in pea (Pisum sativum L.) to evaluate biofortification potential
Source: G3 (Bethesda). 2021 Jul 8;11(9):jkab227. doi: 10.1093/g3journal/jkab227 (PMC8496233; doi:10.1093/g3journal/jkab227)
Supplement: jkab227_Supplementary_Data [file jkab227_supplementary_data.zip › jkab227-suppl_data/GENETICS-G3-2021-402505-s02.pdf]

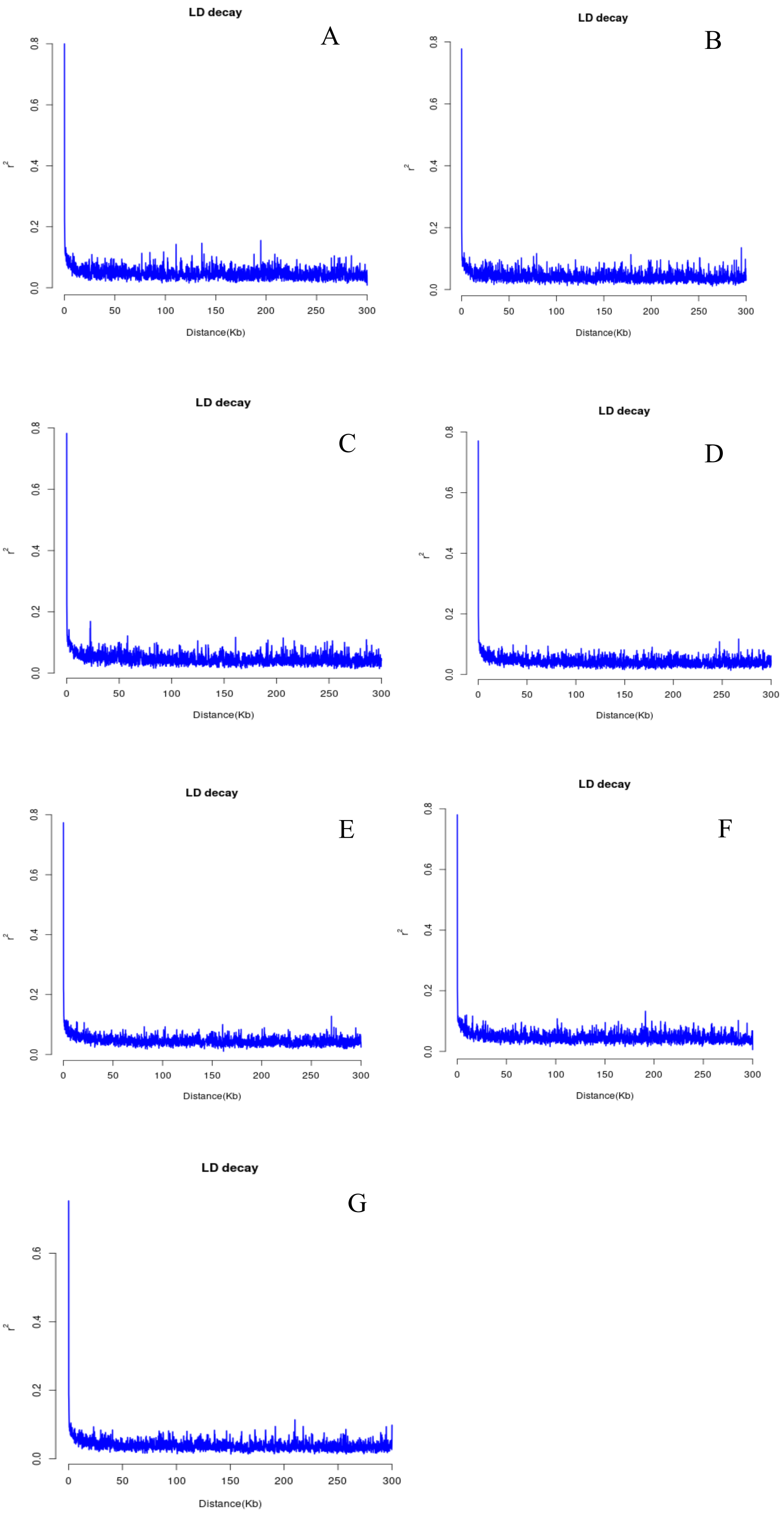


**S-Figure 1.** LD decay plots of chromosome 1-7 (A-G).

MLM.Fe

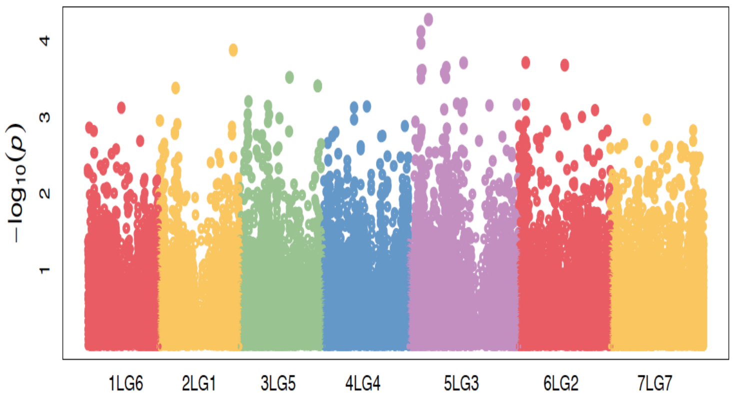


MLM.P

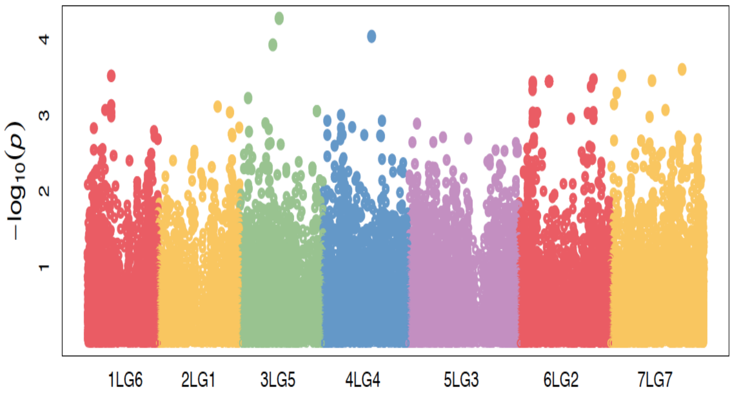


MLM.Zn

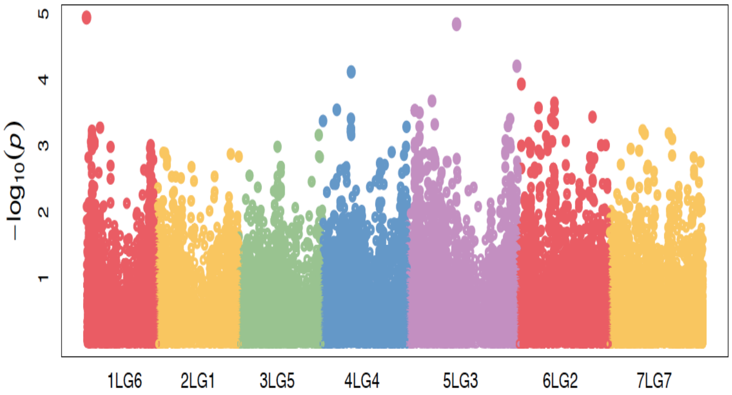

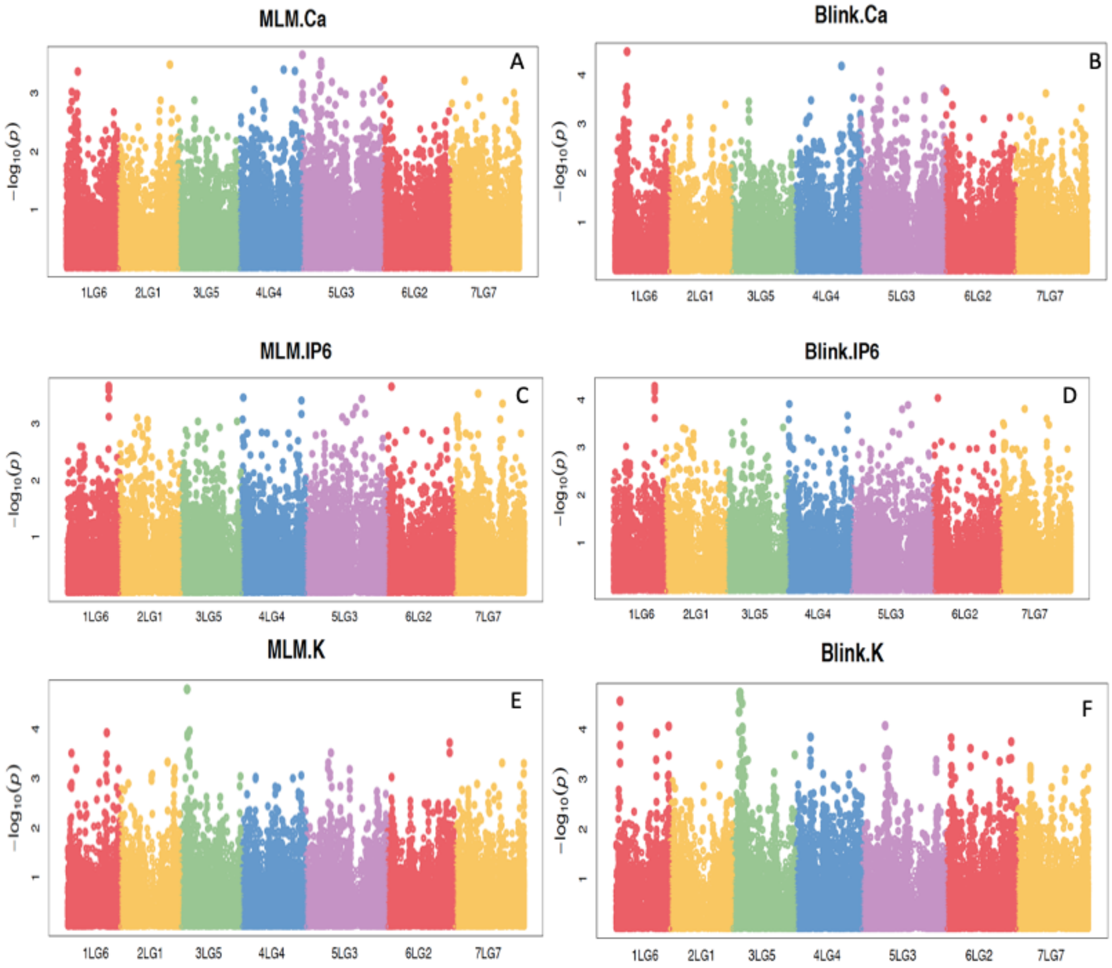


**S-Figure 2.** Manhattan plots for MLM and Blink models of Ca (A, B), IP6 (C, D), and K (E, F) concentration.

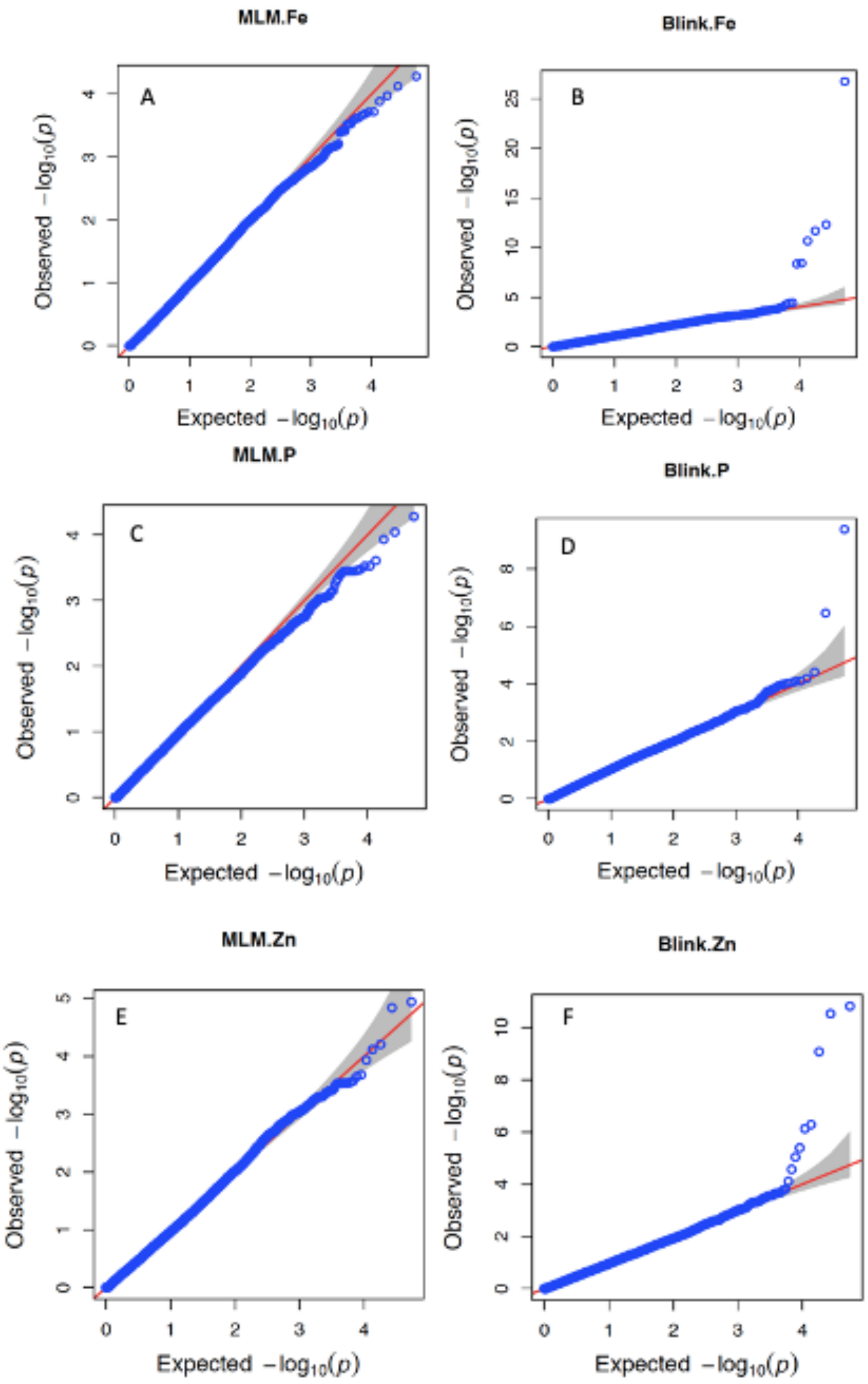


**S-Figure 3.** QQ-Plots for Blink and MLM model for Fe (A, B), P (C, D), and Zn (E, F).

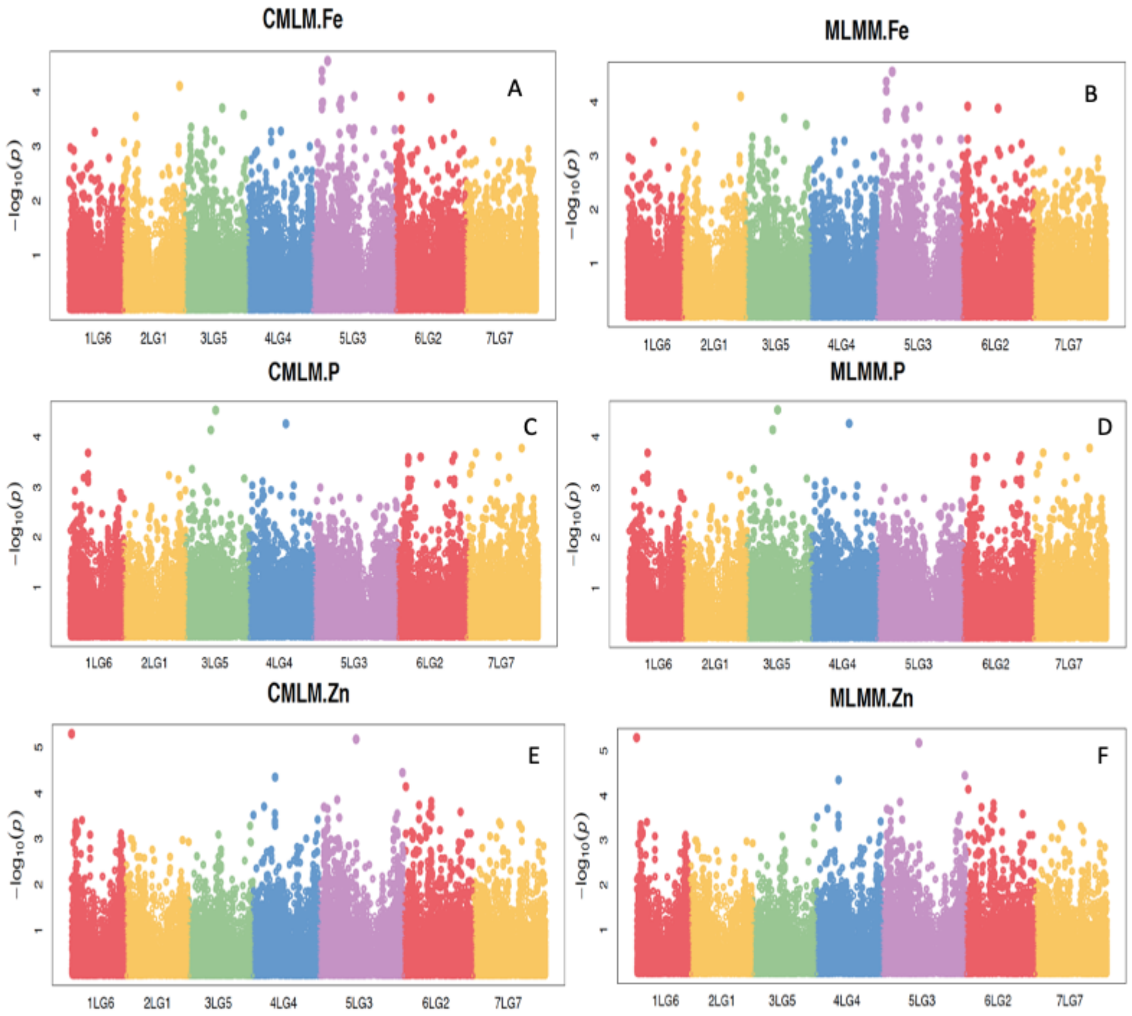


**S-Figure 4.** Manhattan plots for CMLM and MLMM model for Fe (A, B), P (C, D), and Zn (E, F).

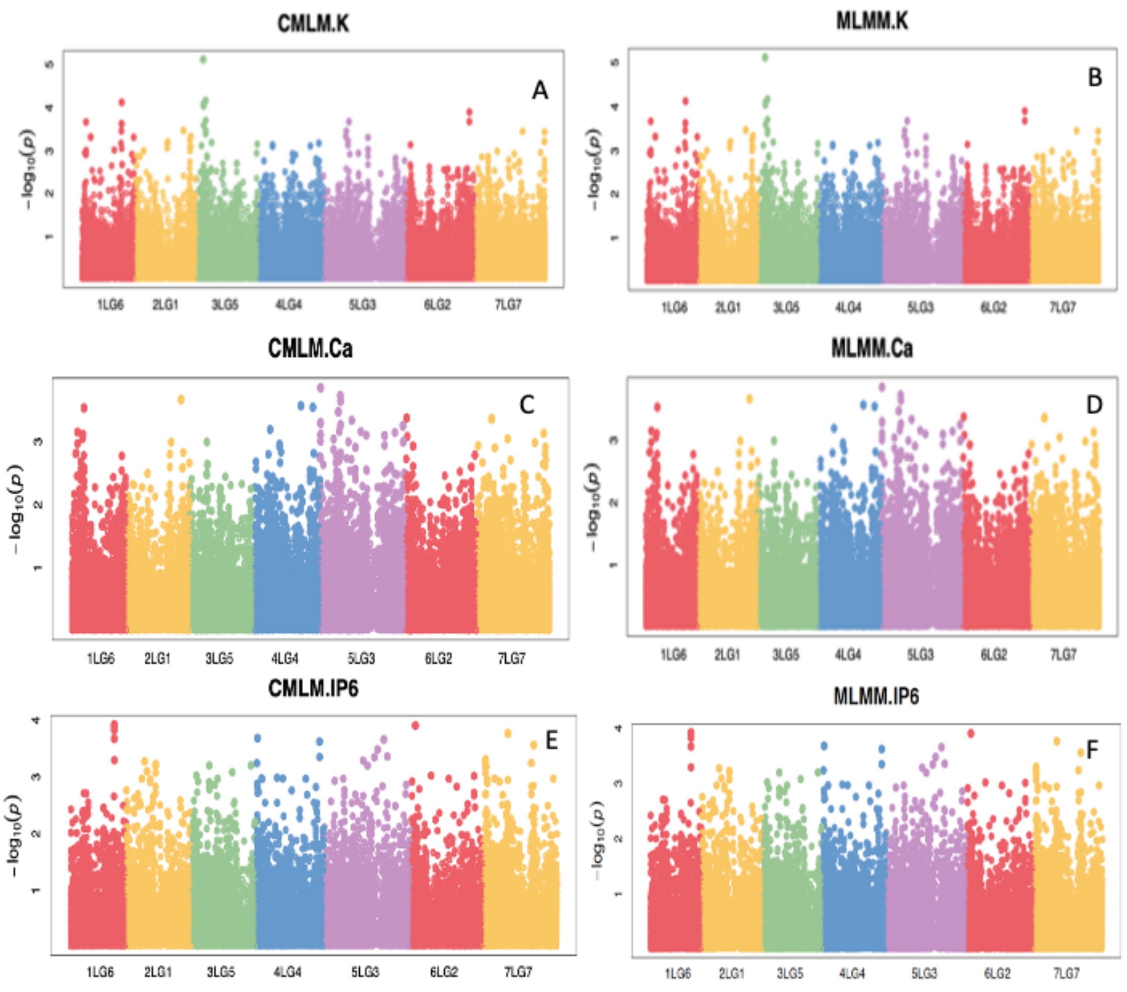


**S-Figure 5.** Manhattan plots for CMLM and MLMM model for K (A, B), Ca (C, D), and IP6 (E, F).

The MLMM model was fit using the following form:

$$\mathbf{y = s_i + S + Q + K + e}$$

where **y** is a vector of the phenotype; $\mathbf{s_i}$ corresponds to markers; **S** is a pseudo quantitative trait nucleotide (QTN); **Q** contains population structure; **K** is a kinship matrix; and **e** is an unobserved vector of residuals, according to the GAPIT user manual (Wang and Zhang, 2020).

The CMLM model was fit using the following form:

$$\mathbf{y = X\beta + Zu + e}$$

where y is a vector of a phenotype; $\boldsymbol{\beta}$ represents unknown fixed effects, including population structure and marker effects; u is a vector of size *s* (number of groups) for unknown random polygenic effects, as well as a group kinship matrix; X and Z are incidence matrices for $\boldsymbol{\beta}$ and **u**, respectively; and e is a vector of random residual effects that are normally distributed with zero mean and covariance (Li et al., 2014).

**S-Table 1**. ANOVA estimates of the effects of accession, date, and replicate on minerals and IP6 concentrations

| | Fe_Df | Fe | Zn_Df | Zn | P_Df | P | K_Df | K | Ca_Df | Ca | IP6_Df | IP6 |
|---|---|---|---|---|---|---|---|---|---|---|---|---|
| TAXA | 246 | ** | 269 | ** | 267 | ** | 278 | ** | 244 | ** | 184 | NS |
| DATE[a] | 26 | ** | 50 | ** | 45 | ** | 49 | ** | 27 | ** | 28 | ** |
| REP | 2 | NS | 2 | NS | 2 | NS | 2 | * | 2 | * | 2 | NS |

** indicates $p < 0.0001$, * indicates $p < 0.05$, and NS means not a significant source of variation

[a]ANOVA with both Date of digestion and date of analysis resulted in singularity issues, so the most significant date was picked for the ANOVA, as well as for BLUP calculations

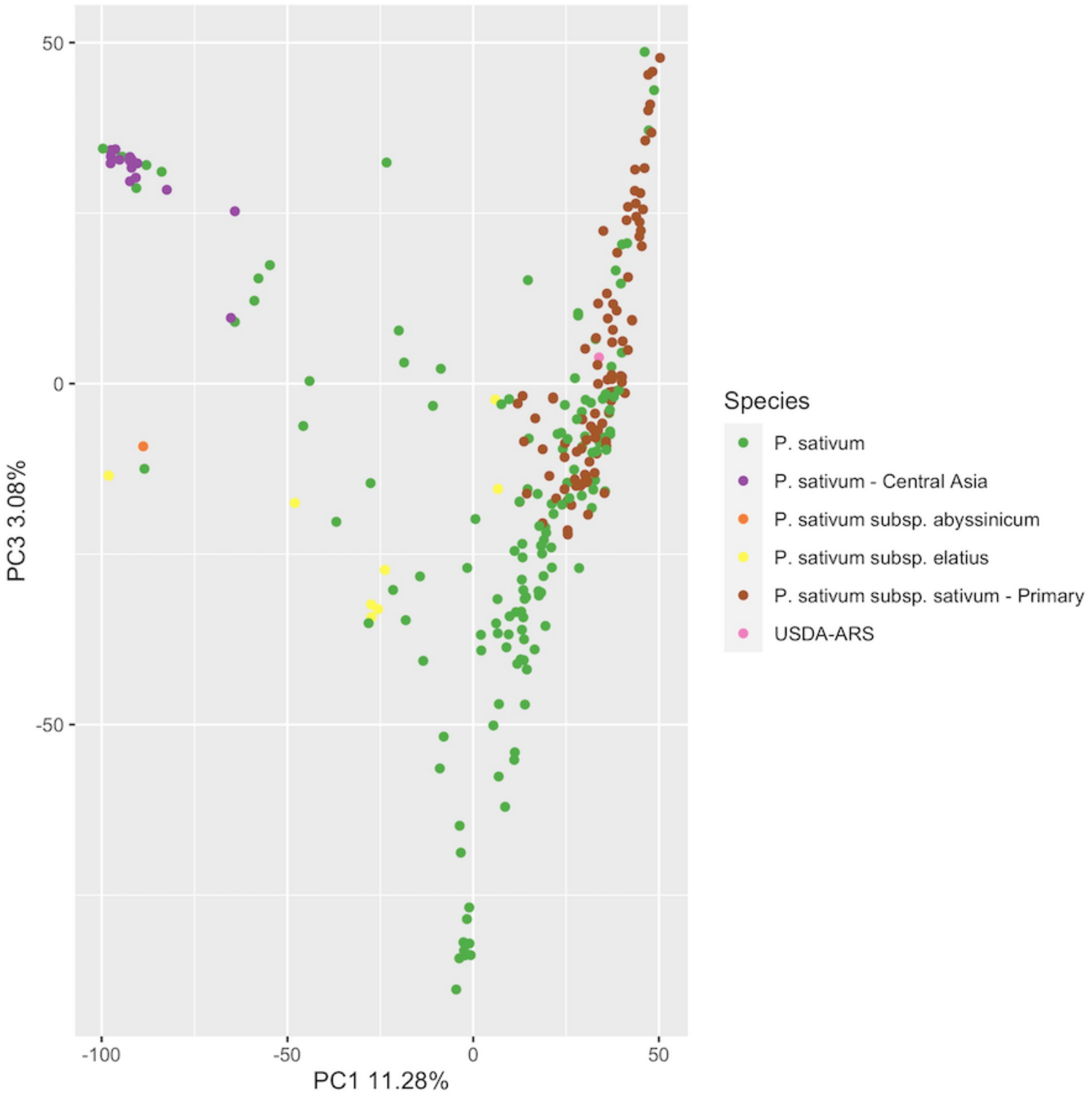


**S-Figure 6.** PCA plot of PC1 vs. PC3 of the study population used for GWAS (n=267). Accessions from the OSU and *P. fulvum* subpopulations were not included in the study population. Accessions with unavailable population information were labelled as *P. sativum*.

References:


1. Li, M., Liu, X., Bradbury, P. *et al*. Enrichment of statistical power for genome-wide association studies. *BMC Biol* **12,** 73 (2014). https://doi.org/10.1186/s12915-014-0073-5
2. Wang, J., and Z. Zhang. 2020. Gapit version 3: Boosting power and accuracy for genomic association and prediction [Preprint]. Bioinformatics. doi: 10.1101/2020.11.29.403170
